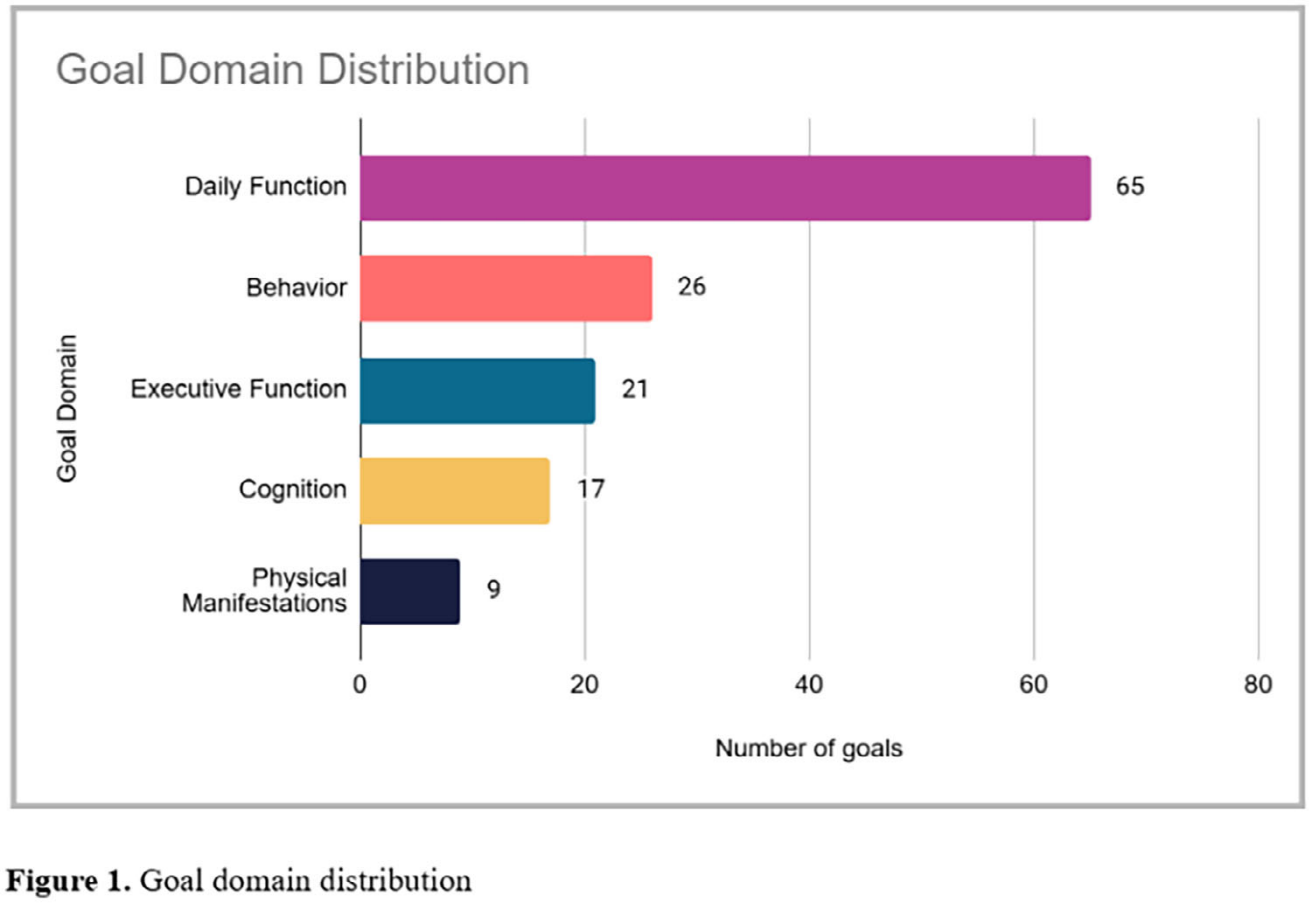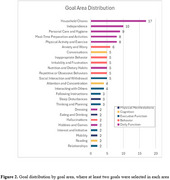# Evaluating the Feasibility of a Personalized Endpoint in Down Syndrome‐associated Alzheimer’s Disease: Inventory‐facilitated Goal Attainment Scaling

**DOI:** 10.1002/alz70861_108133

**Published:** 2025-12-23

**Authors:** Gunes Sevinc, Michelle George, Katie Crespo, Lois Kelly, Hampus Hillerstrom, James Hendrix, Chere Chapman, Kenneth Rockwood

**Affiliations:** ^1^ Ardea Outcomes, Halifax, NS Canada; ^2^ LuMind IDSC Foundation, Woburn, MA USA; ^3^ Eli Lilly and Company, Indianapolis, IN USA; ^4^ Dalhousie University, Halifax, NS Canada; ^5^ Nova Scotia Health, Halifax, NS Canada

## Abstract

**Background:**

Evaluating health outcomes in Down syndrome‐associated Alzheimer’s disease (DS‐AD) is challenging due to variability in baseline function and cognition. Personalized outcome assessments, like Goal Attainment Scaling (GAS), can address this gap and capture treatment responses across varying baseline states and symptom manifestations. However, implementation must be standardized through tools such as goal inventories. Here, we assessed the content validity of the DS‐AD goal inventory (Knox et al., 2020, 2021) and investigated the feasibility and acceptability of inventory‐facilitated GAS as a patient‐centric tool to evaluate treatment response.

**Method:**

We conducted a prospective, 16‐month, non‐interventional study using the DS‐AD goal inventory to facilitate GAS with caregivers of individuals with Down syndrome. The inventory included 58 goal areas distributed across behavior, cognition, daily function, executive function, and physical manifestation domains. The content validity of the goal inventory was assessed through a qualitative analysis of goal scales and alignment with the existing inventory. Goal count, goal scale completeness, and interview durations were used as feasibility indicators. An end‐of‐study survey evaluated acceptability.

**Result:**

Forty‐six caregivers set 3 goals with 5‐levels each and assessed goal attainment at the 3 (*n* =45) and 16‐month follow‐ups (*n* =43). Mean interview times were 38.6 (±10.4) minutes for goal‐setting and 17.9 (±9.5) and 14.5 (±4.7) minutes for 3‐ and 16‐month follow‐ups. Out of 138 goals, 117 were initially selected from the inventory. The qualitative analysis indicated that the majority of the goals were from the Daily Function (*n* =65) and Behavior (*n* =26) domains (Figures 1&2), and that the inventory covered 125 (91%) goals. A qualitative analysis of the remaining goals (*n* =13) revealed diet and physical activity as additional goal areas. Survey results (*n* =33) indicated that caregivers had positive experiences with GAS (*n* =31), found their goals meaningful (*n* =31), valued improved clinician communication (*n* =30), and gained new perspectives and knowledge (*n* =10).

**Conclusion:**

Our findings indicate GAS is feasible and acceptable to caregivers, and the DS‐AD goal inventory comprehensively reflects patient priorities. Additional gaps identified led to inventory enhancements, resulting in a more comprehensive resource to standardize GAS implementation in DS‐AD studies.